# Peer review of "A Novel Salt Inducible Kinase 2 Inhibitor, ARN-3261, Sensitizes Ovarian Cancer Cell Lines and Xenografts to Carboplatin"

_cancers, 2021, doi:10.3390/cancers13030446_

Round 1

Reviewer 1 Report

Bottom line is they responded to all points. While I would love to see some other studies it is beyond the scope of the current paper and the current paper should be accepted for publication.

Reviewer 2 Report

Regarding this comment:

Cisplatin resistant cell lines were obtained from Dr. Anil Sood’s lab. Although platinum resistance was induced in this cell line with cisplatin, it has been shown to be resistant to carboplatin as well.

Do the authors have evidence the cell line is resistant to carboplatin, maybe include as supplementary or a reference

This manuscript is a resubmission of an earlier submission. The following is a list of the peer review reports and author responses from that submission.

Round 1

Reviewer 1 Report

Review

Manuscript presents an interesting investigation concerning inhibition of  Salt-induced kinase 2 (SIK2) by a new inhibitor ARN-3261. Authors presents results of study in clear and logic in a clear and logical way. ARN-3261 presents cytotoxic effect against 7 from 8 investigated ovarian cancer cell lines and against cisplatin resistant cell line as well. Furthermore in presents a synergistic cytotoxic effect with carboplatin. Next authors presents the mechanism of action related to enhanced DNA damage, increased apoptosis and  downregulating of survivin expression. In animal study it also collaborate with carboplatin and inhibit tumor grow in mouse xenograft models. This indicate that this new agent has also ability to breakdown resistant mechanism not only related to single cancer cells but also related to tumor tissue like extracellular matrix expression and dense cellular structure in tumor.

However we should remember about other resistant mechanism like over expression of drug transporters and especially glycoprotein P (P-gp) and breast cancer resistant protein (BCRP). It is possible the ARN-3261 is a substrate for these drug efflux pomps. That I suggest authors (not for the current manuscript), to compare the effectiveness of ARN-3261 against cell lines with low and high level of drug transporters expression.

According to manuscript, the quality of figures should be improved. I suggest to increase the size of figures 1, 2 and 4.

Conclusion is also very modest, it should contain a few sentences more, such as that the inhibitor has an effect both in in vitro model on single cells and in in vivo model on a tumor.

Author Response

The manuscript presents an interesting investigation concerning inhibition of Salt-induced kinase 2 (SIK2) by a new inhibitor ARN-3261. Authors present results of their study in a clear and logical way. ARN-3261 presents cytotoxic effect against 7 from 8 investigated ovarian cancer cell lines and against cisplatin resistant cell line as well. Furthermore it presents a synergistic cytotoxic effect with carboplatin. Next authors present the mechanism of action related to enhanced DNA damage, increased apoptosis and downregulating of survivin expression. In animal study it also collaborate with carboplatin and inhibit tumor grow in mouse xenograft models. This indicate that this new agent has also ability to breakdown resistant mechanism not only related to single cancer cells but also related to tumor tissue like extracellular matrix expression and dense cellular structure in tumor.

However we should remember about other resistant mechanism like over expression of drug transporters and especially glycoprotein P (P-gp) and breast cancer resistant protein (BCRP). It is possible the ARN-3261 is a substrate for these drug efflux pomps. That I suggest authors (not for the current manuscript), to compare the effectiveness of ARN-3261 against cell lines with low and high level of drug transporters expression.

According to manuscript, the quality of figures should be improved. I suggest to increase the size of figures 1, 2 and 4.

Conclusion is also very modest, it should contain a few sentences more, and such as that the inhibitor has an effect both in in vitro model on single cells and in in vivo model on a tumor.

P-gp and BCRP: Arrien Pharmaceuticals, our collaborator and the manufacturer of ARN-3261, had tested the drug efflux in Caco-2 cells (clone C2BBe1). This clone expresses MDR1, BCRP and MRP2 drug transporters. ARN-3261 and ARN-3236 from which ARN-3261 is derived had been tested. The efflux ratios for ARN-3261 and -3236 are 2.47 and >20, respectively. ARN-3261 is classified as negative for efflux while ARN-3236 is positive. Thus, ARN-3236 was replaced by ARN-3261. We revised the introduction to reflect this aspect (Introduction, para 3, lines 68-72).

Quality of figures: All the original figures are saved at 600dpi in TIFF format. When we submitted the figures as a compiled PDF file, we had to downsize the figures in jpg format so the PDF wouldn’t max out the size requirement. We will upload individual figures with better resolution for the final manuscript.

Conclusion: The Conclusion has been rewritten to include more information (lines 406-408).

Reviewer 2 Report

The authors present data regarding the use of ARN-3261 an inhibitor of SIK-2 in ovarian cancer and its ability to potentiate carboplatin's effects in the treatment of OVCA. While this is potentially of interest for at least a subset of patients, the current quality of the data does not allow me to approve for publication in Cancers.

Overall the presentation of data is good, however, unfortunately, short on actual conclusive outcomes. Some of my major concerns are noted below.

The mechanism of action prescribed by the authors, at least with the data currently presented is insufficient to adequately support their conclusions:

  1. The majority of the data presented is descriptive.  While I agree that there does appear to be some mild synergism in some lines the data presented is not convincing (for example when describing a platinum resistant line the CI index is 0.5 but the actual IC50 is still QUITE high not able to be achieved in vivo!). Additionally, apoptosis is increased in SOME of the cell lines the actual degree of change is somewhat minimal.  Ideally, the authors would identify cell lines with overexpression of SIK-2 and those with normal to low levels of SIK-2 (especially given SIK-2 is only over-expressed in 30% of all OVCA) and demonstrate differential effects of their drug.  if they do NOT see a difference then this would need to be explained mechanistically!
  2. The only mechanistic data the authors attempt to present is limited data regarding survivin.  As the current immunoblots are presented, I unfortunately would disagree with their conclusion.  These WB are not of sufficient quality that I would determine that there is indeed a true difference between the various conditions.  They would need to be repeated and additional KD would need to be considered in this setting for me to determine if there is indeed a connection here!
  3. The KD data for SIK-2 is itself problematic.  While there may be statistical significance to the apoptosis changes, I am far from impressed for a control to combo apoptosis change of ~5%.  Again, this would ideally be repeated in cell lines that have overexpressed SIK-2as compared to those that do not and THEN conclusions might be able to be identified.

Author Response

The authors present data regarding the use of ARN-3261 an inhibitor of SIK-2 in ovarian cancer and its ability to potentiate carboplatin's effects in the treatment of OVCA. While this is potentially of interest for at least a subset of patients, the current quality of the data does not allow me to approve for publication in Cancers.

Overall the presentation of data is good, however, unfortunately, short on actual conclusive outcomes. Some of my major concerns are noted below.

The mechanism of action prescribed by the authors, at least with the data currently presented is insufficient to adequately support their conclusions:

  1. The majority of the data presented are descriptive. While I agree that there does appear to be some mild synergism in some lines the data presented is not convincing (for example when describing a platinum resistant line the CI index is 0.5 but the actual IC50 is still QUITE high not able to be achieved in vivo!). Additionally, apoptosis is increased in SOME of the cell lines the actual degree of change is somewhat minimal. Ideally, the authors would identify cell lines with overexpression of SIK-2 and those with normal to low levels of SIK-2 (especially given SIK-2 is only over-expressed in 30% of all OVCA) and demonstrate differential effects of their drug.  If they do NOT see a difference then this would need to be explained mechanistically!

Our cell viability assay data indicate that SIK2 inhibition enhances carboplatin sensitivity in ovarian cancer cells that are relatively sensitive to carboplatin and relatively resistant to carboplatin (revised lines 86-94). While we appreciate that some carboplatin IC50’s are high even in the presence of ARN-3261, there is a significant increase in sensitivity to carboplatin in 7 of 8 cell lines and a synergistic interaction between carboplatin and ARN-3261 documented in at least 4 cell lines.

SIK2 is expressed in most tissues. While the therapeutic index for a SIK2 inhibitor may depend upon the difference in SIK2 expression between cancers and normal tissues, to date it appears that ARN-3261 has little toxicity for normal tissues in mice or dogs, prompting the initiation of a phase I human trial.

All of the ovarian cancer cell lines and tumor tissues we tested express different levels of SIK2. We had published data indicating that endogenous SIK2 protein expression correlates inversely with IC50 values of ARN-3236 in 10 ovarian cancer cell lines (J Zhou, et al., 2017 Clinical Cancer Research), including 7 of the 8 cell lines used in the present study. ARN-3236 is another selective SIK2 inhibitor from which ARN-3261 was derived by a single sulfone substitution. When SIK2 expression was compared in different ovarian cancer cell lines by western blot in our earlier paper, strong SIK2 expression was detected in 3 of the 4 cell lines (SKOv3, OVCAR 8, and OC316) that subsequently showed synergistic interaction between carboplatin and ARN-3261 in the present study. Weak SIK2 expression was observed in a cell line that failed to show significant sensitization to carboplatin with ARN-3261 (ES2).  Synergistic inhibition was, however, observed in one cell line with very weak SIK2 expression (IGROV1), suggesting that there is not a perfect correlation between SIK2 expression and potentiation of carboplatin toxicity. With or without a correlation, the mechanism that we propose in this article, where SIK2 inhibition further increases DNA damage, decreases survivin and increases apoptosis, could well depend upon intrinsic properties of the ovarian cancer cells with increased free radical production and impaired DNA repair, rather than on the expression of SIK2. Shutting down SIK2 activity with ARN-3261, independent of baseline SIK2 levels, could decrease DNA repair and decrease survivin, enhancing apoptosis. Complete or nearly complete SIK2 inhibition could be well tolerated by normal cells, but prove lethal for ovarian cancer cells treated with carboplatin.            

  1. The only mechanistic data the authors attempt to present is limited data regarding survivin. As the current immunoblots are presented, I unfortunately would disagree with their conclusion. These WB are not of sufficient quality that I would determine that there is indeed a true difference between the various conditions.  They would need to be repeated and additional KD would need to be considered in this setting for me to determine if there is indeed a connection here!

We agree that more convincing western blots are required. Consequently, we repeated the experiments three times and included OVCAR8-SIK2 knockout cell line. The new figure 3 was added to line 158 and the legend was revised on lines 162-169.  

  1. The KD data for SIK-2 is itself problematic. While there may be statistical significance to the apoptosis changes, I am far from impressed for a control to combo apoptosis change of ~5%. Again, this would ideally be repeated in cell lines that have overexpressed SIK-2as compared to those that do not and THEN conclusions might be able to be identified.

We have repeated apoptosis assays twice using two SIK2 KD cell lines and achieved reproducible results presented in Figure 2B.

Reviewer 3 Report

The manuscript is very good written, although it requires some corrections and revision.

Abstract:

Several sentences / phrases in the Abstract were copied from the Introduction; they should be rewritten.

Results

Figure 1B and 1C: What is the difference between chart B (black line) and chart C (blue line)? Are the experiments performed in the same way (carboplatin only)? If so, why are the IC50 values of carboplatin drastically different in both experiments for the same cell lines? If not (blue chart is with solvent only) why is there such huge influence of a solvent?

Figure 1C: Single concentration of ARN-3261 was completely different in relation to the IC50 value for a certain cell line: A2780 1.15 µM (above IC50 0.8 µM), ES2 1.5 µM (below IC50 0.9 µM), IGROV1 0.75 µM (considerably below IC50 2.8 µM), MD2774 1.15 µM (equal IC50 1.1 µM), OC316 ….missing data, OVCAR3 0.75 µM (below IC50 1.7 µM), OVCAR8 1.15 µM (below IC50 2.1 µM) and SKOv3 1.5 µM (equal IC50 1.4 µM). How do the Authors explain these discrepancies and inconsistencies in the selection of the concentrations for individual cell lines? Please comment and explain in Materials and Methods.

Figure 1D: From my point of view, the effect of the ARN-3261 and carboplatin combination is rather additive, not synergistic. However, the figures are so small that it is difficult to judge. A summary table in the Supplementary Materials will be useful; for Authors' consideration.

Figure 3. Mean values of densitometry +/- SD from 3 independent experiments should be shown. Are the numerical values under the photos normalized to the reference values?

Figure 4. Have p values been corrected for multiple comparisons? If not, please provide the name of the statistical test in the figure caption.

Materials and Methods

The Drugs and Reagents chapter is missing. Information about the purity and patent number should be provided for ARN-3261. In what solvent were ARN-3261 and carboplatin dissolved, and what was the carboplatin stock concentration? Final concentration of solvent should be provided. The source and purity of carboplatin and paclitaxel should be stated.

Growth inhibition assays: What was the initial cell density? How long have cells been incubated with ARN-3261 and / or carboplatin? What was cytotoxic effect in the ATP "Growth inhibition assay"?

Statistical analysis: “All experiments were repeated independently at least twice”. Standard deviation calculated for n = 2 is incorrect. This should be corrected, e.g. “the standard deviation was calculated for experiments performed at least in triplicate”.

Conclusions

“SIk2 inhibitor ARN-3261 enhances sensitivity to both carboplatin-sensitive and resistant ovarian cancer cells.” Do you mean “… ARN-3261 enhances sensitivity to carboplatin of both…”?

Author Contributions:

Does the supply of reagents, even crucial ones, justify authorship? Was it the only contribution of HV and AA to this work?

Author Response

Abstract:

Several sentences / phrases in the Abstract were copied from the Introduction; they should be rewritten.

The Introduction has been rewritten to avoid duplicate sentences (lines 56-64).

Results

Figure 1B and 1C: What is the difference between chart B (black line) and chart C (blue line)? Are the experiments performed in the same way (carboplatin only)? If so, why are the IC50 values of carboplatin drastically different in both experiments for the same cell lines? If not (blue chart is with solvent only) why is there such huge influence of a solvent?

1B. Carboplatin only. Carboplatin is dissolved in water.

1C. Combination test: ARN-3261 is dissolved in DMSO. The vehicle control and carboplatin alone in chart C were supplemented with the equal amount of DMSO to ensure the condition in all three groups was even except for different drug treatment.

Experiments for B and C were performed separately, weeks or even months apart. A2780, ES2 and IGROV1 are the three cell lines that shows relatively big difference in IC50 values between Chart B and C. These three cell lines are fast growing and are particularly challenging for replicating dose-response assays using 96-well plates. Subtle differences in cell dilution, drug dilution, or growing conditions can cause large variations. However, the trend for each repeat experiment that we performed is similar.

Figure 1C: Single concentration of ARN-3261 was completely different in relation to the IC50 value for a certain cell line: A2780 1.15 µM (above IC50 0.8 µM), ES2 1.5 µM (below IC50 0.9 µM), IGROV1 0.75 µM (considerably below IC50 2.8 µM), MD2774 1.15 µM (equal IC50 1.1 µM), OC316 ….missing data, OVCAR3 0.75 µM (below IC50 1.7 µM), OVCAR8 1.15 µM (below IC50 2.1 µM) and SKOv3 1.5 µM (equal IC50 1.4 µM). How do the Authors explain these discrepancies and inconsistencies in the selection of the concentrations for individual cell lines? Please comment and explain in Materials and Methods.

We corrected these values in the legend to Fig. 1 (lines 112-113). Here are the correct concentrations of ARN-3261 that were used in Figure 1C: A2780 0.75 µM, ES2 1.25 µM, IGROV1 1.25 µM, MD2774 1.15 µM, OC316 0.75 µM, OVCAR3 0.75 µM, OVCAR8 1 µM and SKOv3 1 µM. We performed this experiment several times to optimize the concentration. We started using the concentration equal to the IC50 value of ARN-3261 for the single treatment in Chart 1A. But we quickly found out the combination killed too many cells for all cell lines but ES2 and MD2774 and we couldn’t do curve fitting for carboplatin dose-response treatment. Therefore, we reduced the ARN-3261 concentration. And the concentrations shown above can shift the carboplatin dose-response curve to the left, indicating improved drug responses. We have added a description of this approach to Material and Methods (4.3).

Figure 1D: From my point of view, the effect of the ARN-3261 and carboplatin combination is rather additive, not synergistic. However, the figures are so small that it is difficult to judge. A summary table in the Supplementary Materials will be useful; for Authors' consideration.

Table S1 has been added to supplementary materials and noted on lines 93 and 411.

Figure 3. Mean values of densitometry +/- SD from 3 independent experiments should be shown. Are the numerical values under the photos normalized to the reference values?

We repeated the WB and included OVCAR8-SIK2 KO cell line (suggested by Reviewer 2) and measured the band intensity. The changes in survivin levels is normalized to GAPDH.  The averages of three replicates were plotted under the WB and compared using one-way ANOVA. New figure 3 was added to revised manuscript.

Figure 4. Have p values been corrected for multiple comparisons? If not, please provide the name of the statistical test in the figure caption.

P values were calculated using One-way ANOVA for multiple comparisons. The method of analysis has been added to figure legend (lines 194-195).

Materials and Methods

The Drugs and Reagents chapter is missing. Information about the purity and patent number should be provided for ARN-3261. In what solvent were ARN-3261 and carboplatin dissolved, and what was the carboplatin stock concentration? Final concentration of solvent should be provided. The source and purity of carboplatin and paclitaxel should be stated.

The information has been added to Materials and Methods section as 4.1 Reagents.

Growth inhibition assays: What was the initial cell density? How long have cells been incubated with ARN-3261 and / or carboplatin? What was cytotoxic effect in the ATP "Growth inhibition assay"?

Statistical analysis: “All experiments were repeated independently at least twice”. Standard deviation calculated for n = 2 is incorrect. This should be corrected, e.g. “the standard deviation was calculated for experiments performed at least in triplicate”.

Growth inhibition assays: What was the initial cell density? How long have cells been incubated with ARN-3261 and / or carboplatin?

The initial cell density was 2,000 cells/well in 96-well plates. Cells were incubated with different treatments for 96 hrs. This information was indicated in figure 1A legend (lines 106-107).

What was cytotoxic effect in the ATP "Growth inhibition assay"?

This is the CellTiter-Glo luminescent cell viability assay by Promega. It measures mitochondrial ATP levels to determine cell viability. The luminescence signals at different doses were normalized to untreated vehicle control (%). The reverse sigmoidal curve shows the cytotoxic effect with decreased cell viability.

Statistical analysis: “All experiments were repeated independently at least twice”. Standard deviation calculated for n = 2 is incorrect. This should be corrected, e.g. “the standard deviation was calculated for experiments performed at least in triplicate”.

In each cell viability experiment, for each cell line and each treatment, we have 6 replicates (six 96 well plates). We repeat each experiment at least twice. So the standard deviation was calculated from at least 12 replicates from two independent experiments. All other experiments were set up in triplicate.

Conclusions

“SIk2 inhibitor ARN-3261 enhances sensitivity to both carboplatin-sensitive and resistant ovarian cancer cells.” Do you mean “… ARN-3261 enhances sensitivity to carboplatin of both…”?

Yes. We revised the sentence. (lines 406-408)

Author Contributions:

Does the supply of reagents, even crucial ones, justify authorship? Was it the only contribution of HV and AA to this work?

For HV and AA, they not only provided the critical reagents, but also helped in formulating the study and revised the manuscript critically for important intellectual content.

Reviewer 4 Report

This is a very nice piece of work from the authors and targeting a cancer that has a dreadful mortality. The work is well presented. I have a number of comments.

It is not clear why the previous publication from the authors looking at ARN-3236 was not pursued with carboplatin. Apart from a line in the introduction ”For clinical use, ARN-3261 appeared more promising than the initial candidate compound ARN 3236 which exhibited low cell permeation and high P-gp efflux”. Is there evidence supporting this? ARN-3236 seemed to perform well with paclitaxel in that publication and also in xenografts. It is surprising that both drugs were not examined by the authors in the same paper and the combination of drugs. It raises concerns that this compound could meet the same fate?

The rationale for the cell lines is not explained, given that the authors are targeting high grade serous, the A2780 model does not represent this subtype.

It is unclear why the authors chose a cisplatin resistant cell line and not a carboplatin resistant model which would fit with the rest of the data they are showing.

The compound clearly has activity and warrants further investigation but I would like to see the response to carboplatin and paclitaxel in parallel and in combination, in particular given the response to paclitaxel in their previous publication, is this data available?

A number of recent papers have been published exploring the role of SIK2 in ovarian cancer and should be included in this publication.

Is there a rationale for the number of mice studied in each group?

The authors treat the mice with a number of other drugs including olaparib, had these been testing in vitro?

Is there a hypothesis as to why it is only expressed in 30% of high grade serous?

Author Response

This is a very nice piece of work from the authors and targeting a cancer that has a dreadful mortality. The work is well presented. I have a number of comments.

It is not clear why the previous publication from the authors looking at ARN-3236 was not pursued with carboplatin. Apart from a line in the introduction ”For clinical use, ARN-3261 appeared more promising than the initial candidate compound ARN 3236 which exhibited low cell permeation and high P-gp efflux”. Is there evidence supporting this? ARN-3236 seemed to perform well with paclitaxel in that publication and also in xenografts. It is surprising that both drugs were not examined by the authors in the same paper and the combination of drugs. It raises concerns that this compound could meet the same fate?

We identified SIK2 as a kinase modulating paclitaxel sensitivity in ovarian cancer cells through a high-throughput kinome siRNA screen. And our initial work was focused on improving paclitaxel sensitivity. Arrien Pharmaceuticals developed a family of SIK2 kinase inhibitors, and ARN-3236 was one of the best SIK2 selective inhibitors. We tested the combination of ARN-3236 and paclitaxel and published the paper (J Zhou, et al., Clinical Cancer Research) in 2017. However, ARN-3236 is the substrate of P-glycoprotein and drug resistance is likely to be encountered. ARN-3261 is derived from 3236 with sulfone substitution and is not subject to P-glycoprotein transporter. The drug efflux assays were performed by the company to evaluate if 3236 and 3261 are substrates of P-gp. ARN-3261 turned out not to be transported by P-gp whereas 3236 did. ARN-3261 replaced 3236 and Arrien stopped the production of 3236. We never get the chance to fully test carboplatin with ARN-3236. Here, we tested ARN-3261 with paclitaxel in animal studies and it shows similar effects as ARN-3236 did.

The rationale for the cell lines is not explained, given that the authors are targeting high grade serous, the A2780 model does not represent this subtype.

Our research is mainly focused on drug resistance in high grade serous ovarian cancer. But as we tested this drug on a variety of cell lines, we found it has effects on high grade and low grade serous cancer cell lines, and breast cancer cell lines regardless of TP53 status and hormone receptors (data not shown). Even though the origin of A2780 is arguable, we still included it in the initial test. However, for critical experiments such as DNA damage and animal xenograft studies, we did not use this cell line.

It is unclear why the authors chose a cisplatin resistant cell line and not a carboplatin resistant model which would fit with the rest of the data they are showing.

Cisplatin resistant cell lines were obtained from Dr. Anil Sood’s lab. Although platinum resistance was induced in this cell line with cisplatin, it has been shown to be resistant to carboplatin as well.

The compound clearly has activity and warrants further investigation but I would like to see the response to carboplatin and paclitaxel in parallel and in combination, in particular given the response to paclitaxel in their previous publication, is this data available?

Unfortunately, we did not test the combination of ARN-3261 and paclitaxel extensively and we do not have data for three drugs in parallel and in combination.

A number of recent papers have been published exploring the role of SIK2 in ovarian cancer and should be included in this publication.

We added several recent SIK2 papers in the Introduction section.

Is there a rationale for the number of mice studied in each group?

Dr. Neely Atkinson, a professor of biostatistics, provided the approximation for our animal studies: Assuming we are comparing two groups which are normally distributed with a common variance between them, 5 mice per group allows us to detect a shift in the mean of 2.0 standard deviations (STD); 10 mice is sufficient for a shift of 1.3 STDs; and 12 mice is sufficient for a shift of 1.2 STDs. Suppose we have 10 mice per group and compare tumor size at 60 days. If the mean value of tumor size for control group is 1 cm and the STD is 0.1 cm, we can detect a shift of 1.3 STD, we can detect a decrease to 1.0-1.3x0.1=0.87. If the STD is 0.2 cm, we can detect a decrease to 1.0-1.3x0.2=0.74. Based on the statistical approximation, we used 10 mice per group to be able to detect a significant difference between control and different treatment groups.

The authors treat the mice with a number of other drugs including olaparib, had these been testing in vitro?

The drugs tested in this paper were ARN-3261, carboplatin and paclitaxel. We corrected it in Results section 2.5. (lines 236)

Is there a hypothesis as to why it is only expressed in 30% of high grade serous?

It is unclear why SIK2 is only overexpressed in a fraction of high grade serous cases. This AMPK family kinase is expressed in most tissues and is important for cell survival. When we knocked down this kinase using siRNA, we saw growth inhibition, and vice versa. Overexpression of SIK2 definitely has survival advantage for cancer cells, and it is related to poorer survival rate. However, the underlying mechanisms are still unclear, further investigations are obviously needed.
