# Supplementary material for: A Novel Salt Inducible Kinase 2 Inhibitor, ARN-3261, Sensitizes Ovarian Cancer Cell Lines and Xenografts to Carboplatin
Source: Cancers (Basel). 2021 Jan 25;13(3):446. doi: 10.3390/cancers13030446 (PMC7865895; doi:10.3390/cancers13030446)
Supplement: Supplementary file 1 [file cancers-13-00446-s001.pdf]

cancers

MDPI

Supplement Materials

# A Novel Salt Inducible Kinase 2 Inhibitor, ARN-3261, Sensitizes Ovarian Cancer Cell Lines and Xenografts to Carboplatin

**Dengxuan Fan, Hailing Yang, Weiqun Mao, Philip J. Rask, Lan Pang, Congjian Xu, Hariprasad Vankayalapat, Ahmed A. Ahmed, Robert C. Bast Jr. and Zhen Lu**

**Table S1.** The combinatorial effect of carboplatin and ARN-3261.

| | Combination Ratio [Carbo]:[ARN-3261] | Combination Index at the Effect Level of 95% |
|---|---|---|
| IGROV1 | 3:1 | 0.75 |
| OC316 | 2:1 | 0.19 |
| OVCAR8 | 4:1 | 0.59 |
| SKOv3 | 12:1 | 0.92 |

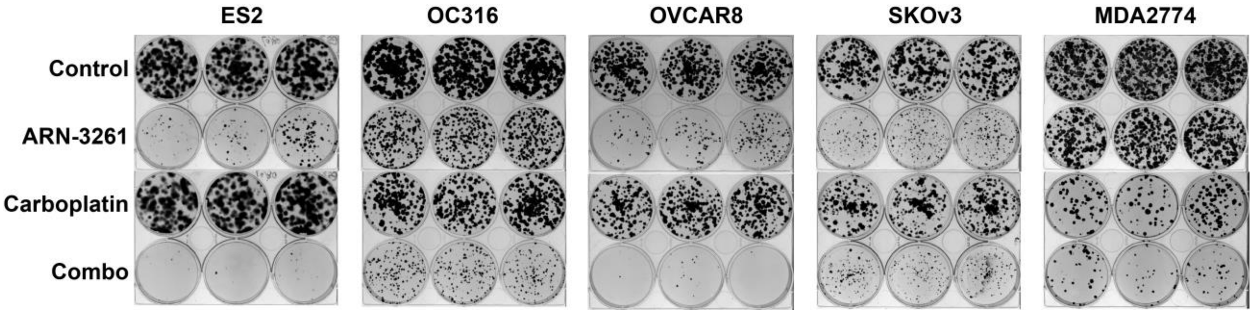


**Figure S1.** ARN-3261 enhances carboplatin-induced inhibition of clonogenic growth. 400 OVCAR8 or SKOv3 ovarian cancer cells were seeded in 6-well plates in normal culture medium for 24 hrs. Cells were then treated with diluent, ARN-3261 (ES2 2.2 μM, OC316 2.5 μM, OVCAR8 2.3 μM, SKOv3 3.5 μM and MDA2774 2.5 μM), carboplatin (ES2 3.3 μM, OC316 3.0 μM, OVCAR8 4.0 μM, SKOv3 2.0 μM and MDA2774 3.0 μM) or both in triplicate for another 12-14 days.

**OC316**

**Repeat 1 and 2** **Long exposure**

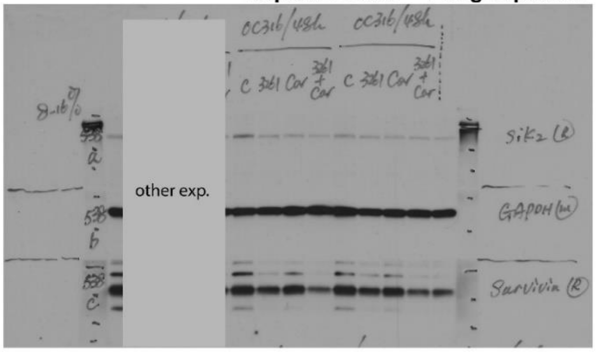


**Short exposure**

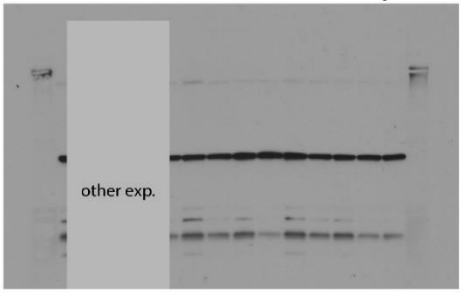


**Repeat 3** **Long exposure**

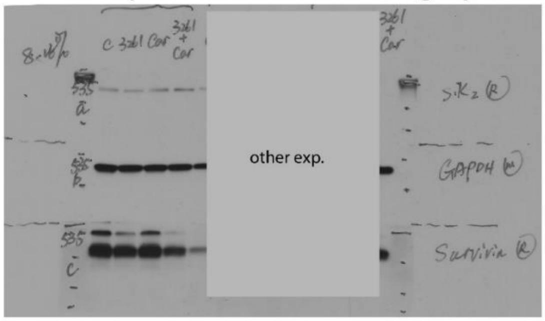


**Short exposure**

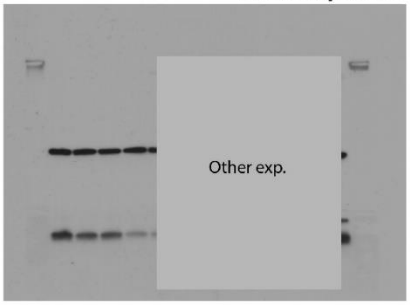

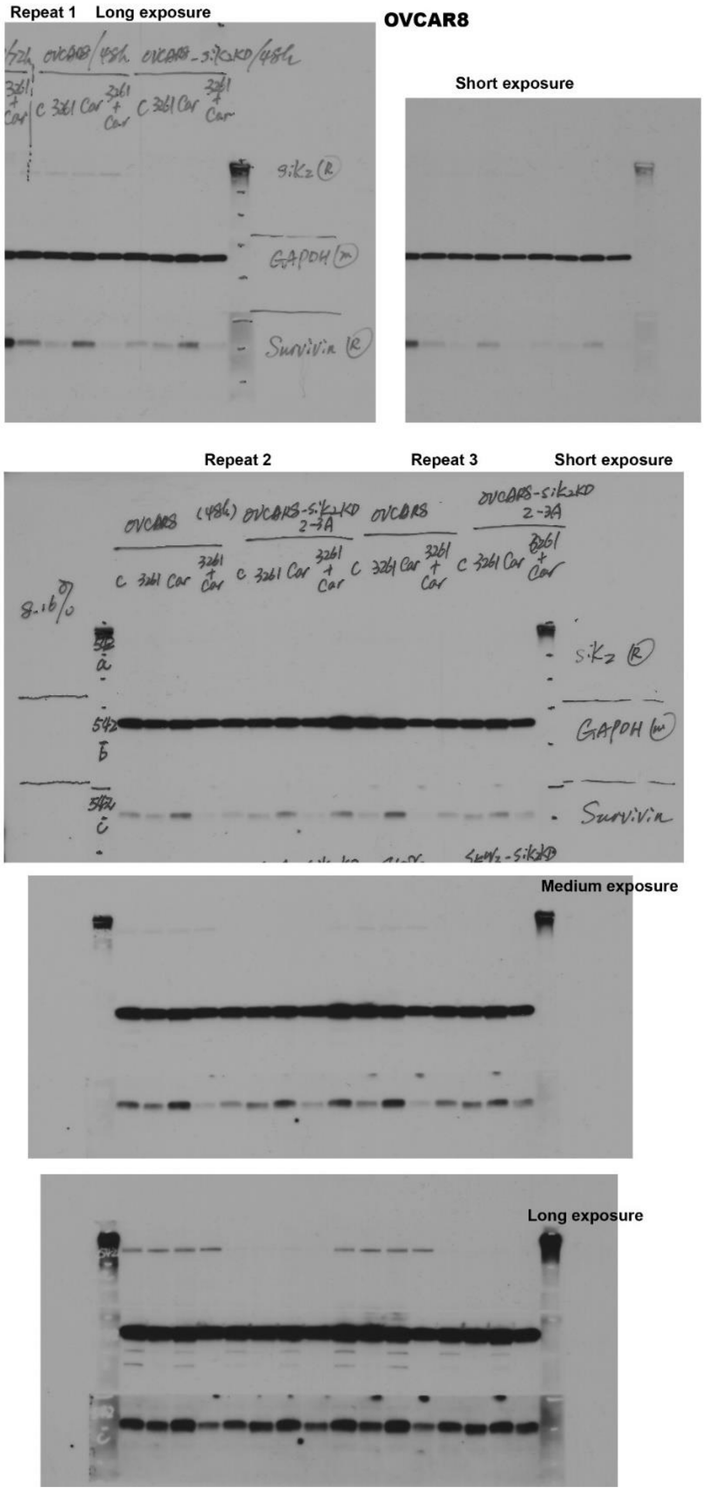


Figure S2: The uncropped western blotting figures.
